# Minilaparoscopic versus single incision cholecystectomy for the treatment of cholecystolithiasis: a meta-analysis and systematic review

**DOI:** 10.1186/s12893-017-0287-x

**Published:** 2017-08-22

**Authors:** Xuan Tan, Guobin Wang, Yong Tang, Jie Bai, Kaixiong Tao, Lin Ye

**Affiliations:** 1 Department of Ophthalmology, Union Hospital, Tongji Medical College, HuaZhong University of Science and Technology, Wuhan, 430022 China; 2 Department of Gastrointestinal Surgery, Union Hospital, Tongji Medical College, HuaZhong University of Science and Technology, Wuhan, 430022 China; 3 Department of Hepatobiliary Surgery, Union Hospital, Tongji Medical College, HuaZhong University of Science and Technology, Wuhan, 430022 China

## Abstract

**Background:**

Over the past decade, mini-laparoscopic cholecystectomy (MLC) and single-port laparoscopic cholecystectomy (SILC) have been the two main successful mini-invasive surgical interventions for the treatment of cholecystolithiasis since the advent of laparoscopic cholecystectomy (LC). In this study, we conducted a meta-analysis to compare the two treatment alternatives.

**Methods:**

We searched PubMed, CNKI and the Cochrane library for trials that compared MLC and SILC. Risk difference (RD) and mean difference (MD) were calculated with a 95% confidence interval (CI).

**Results:**

Four randomized controlled trials (RCTs) and 2 non-randomized comparative studies (nRCSs) involving 2764 patients were identified. A longer operating time (MD -10.49; 95% CI -18.10, −2.88; *P* = 0.007) and a shorter wound length (MD 3.65; 95% CI 0.51, 6.78; *P* = 0.02) were found to be associated with SILC compared with MLC. No significant differences were revealed in conversion, hospital stay, pain relief and cosmetic results. Although a lower incidence of complications was observed with MLC (8.2%) compared with SILC (15.9%), but the difference was not statistically significant (RD -0.06; 95% CI -0.12, 0.00; *P* = 0.07).

**Conclusions:**

MLC has an advantage over SILC in terms of operating time rather than hospital stay, pain relief, cosmetic results. Though conversion and complication rates were higher with SILC, there existed no statistically differences in the two measures between the two procedures. Whether MLC confers any benefits in terms of conversion or complications still warrants further studies.

**Electronic supplementary material:**

The online version of this article (doi:10.1186/s12893-017-0287-x) contains supplementary material, which is available to authorized users.

## Background

Since its introduction at the end of the 1980s [1], LC rapidly has become the best choice for surgical removal of the gallbladder for quicker postoperative recovery and less complications, among others. In the development of laparoscopic technique, minimal invasiveness has been critical for minimizing tissue trauma, reducing postoperative pain and improving cosmetic results [2]. Among cholecystectomy procedures that entail smaller and fewer incisions, MLC [3, 4] and SILC [5, 6] are mostly used.

It had been reported that SILC could achieve better pain relief and excellent cosmetic result when compared to LC [7]. Nonetheless, reports were scanty on the advantages of MLC over LC except for the cosmetic result (small incisions) [8]. Moreover, though multiple studies compared MLC with LC and SILC with LC, so far, no meta-analysis compared MLC with SILC. In this study, we, by conducting a meta-analysis and systemic review, compared the two surgical interventions for the treatment of cholecystolithiasis in terms of their advantages and drawbacks.

## Methods

### Literature research

We searched the following databases: PubMed, Elsevier, Wiley Online Library and the Cochrane Library (up to 30 Dec. 2016), by using the terms ‘LESS’, ‘SILC’, ‘SILS’, ‘single port’, ‘single incision’, ‘single access’, ‘single site’, ‘minilaparoscopic’, ‘microlaparoscopic’, ‘needlescopic’ and ‘scareless’. No effort was made to retrieve any unpublished studies. The ‘related article’ function was utilized to expand the search. The references from the included trials and meta-analyses were searched for additional trials. The study protocol was approved by the Ethics Committee of Union Hospital, Tongji Medical College, Huazhong University of Science and Technology, Wuhan, China. The patients’ personal information was encrypted or made anonymous prior to analysis.

### Definitions

MLC studies were defined as researches including (1) needlescopic (≤ 3 mm at 2-3 sites) and (2) microlaparoscopic (2-3 ports ≥3 mm and <5 mm). LC studies that involved three or two ports were excluded.

SILC was defined as laparoscopic excision of the gallbladder performed through a single trans-umbilical incision using either a multiport device or different individual ports through the same single skin incision.

LC was defined as a conventional 4-ports laparoscopic cholecystectomy using two 10 mm ports and two 5 mm ports.

### Inclusion and exclusion criteria and study selection

Eligible trials that compared MLC and SILC were included, irrespective of blinding, language, sample size or randomization. Studies were considered for inclusion if: they reported at least one of the outcomes covered by this meta-analysis and data were extractable. Studies were excluded if: they did not report any outcome of interest or their data could not be extracted.

Two authors (Yong T and Jie B) evaluated the titles and abstracts of the studies retrieved online. Those that were deemed irrelevant were excluded and full-text version of potentially eligible articles was collected. Inclusion decision was made independently by three reviewers (Yong T, Jie B and Lin Y). If more than two of them agreed on the inclusion of a study, the study was included in this meta-analysis.

### Data extraction

Data from included trials were extracted by Yong T, then Jie B checked the data. Lin Y would weigh in to resolve any disagreements that might arise. An intention-to-treat analysis was performed.

General descriptive data (such as gender, age, sample size, running time, etc.) were extracted from each trial included. Outcomes, including operating time, hospital stay, pain relief, cosmetic scores, and complications were recorded whenever possible. Moreover, the data on methods employed in the trials used were collected for bias analysis.

### Assessment of methodological quality of bias risk of included studies

Cochrane Collaboration’s tool [9] was used to assess the risk of bias for RCTs and random allocation, allocation concealment or blinding was considered as a low risk if they were positively stated, and a high risk if they were not clearly mentioned. Outcomes (including follow up data) were considered low risk for trial registration clearly mentioned and no data withdraw or deficiency, and a high risk were considered for others.

The Newcastle-Ottawa Scale (NOS) [10] was used to assess the risk of bias for nRCSs (Additional file 1). The study was deemed of high quality for it registered a score > = 6.

### Statistical analysis

Meta-analysis was carried out by using two software packages: the Review Manager Ver. 5.3 (The Cochrane Collaboration, the Nordic Cochrane Centre, Copenhagen, Denmark) and MetaAnalyst Beta 3.21 (Tufts Medical Center, Boston, USA).

Quantitative statistical analysis for dichotomous variables was conducted by using risk difference (RD) [9] for binary outcomes with too many zero events in both arms. Mean difference (MD) [9] was used as the summary statistic for quantitative statistical analysis of continuous variables. RD and MD values were reported with 95% confidence intervals (CI). A *P* < 0.05 was considered to be statistically significant. Where studies reported continuous data as medians plus ranges, the mean and standard deviation were calculated using the methods described in the Cochrane handbook [9]. Funnel plots [11] were used for investigating publication bias.

Statistical heterogeneity was determined using the χ2 test [12], with a *P* < 0.05 indicating statistically significant heterogeneity. Clinical heterogeneity was tested by means of the I^2^ value [13]; a value exceeding 50% was indicative of clinical heterogeneity. If heterogeneity was found, random-effects [14] analysis was performed; otherwise the results of fixed-effects [15] analysis were presented. If excessive heterogeneity occurred, data were rechecked first and then adjusted. Sensitivity analyses were performed and extreme outliers were excluded. Subgroup analyses were conducted to identify the causes of heterogeneity.

If meta-analysis of the data was not possible because of heterogeneity in measurement methods, descriptive (qualitative) analyses were carried out by reporting the number of studies that found a significant difference between the procedures.

## Results

### Characteristics of included studies

**Fig. 1:**
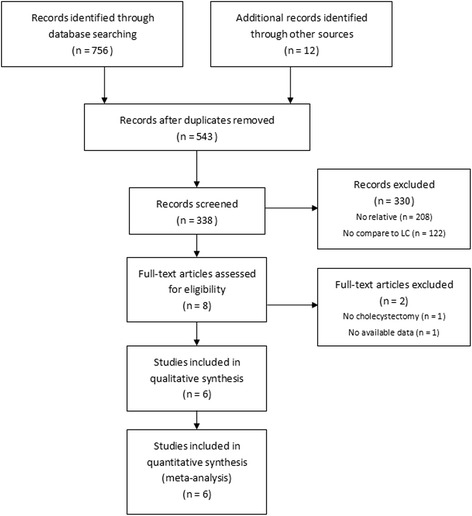
Flow diagram of included studies

Eight publications were full-text reviewed independently by two reviewers (Yong T and Jie B). Differences between Yong T and Jie B were resolved by comparing notes with Lin Y. One study [16] was excluded for unavailability of data and one study [17] was removed for hysterectomy. Eventually, 6 publications (4 RCTs [18–21] and 2 nRCSs [22, 23]) involving 2764 patients were included for further evaluation (Table 1). Then a flow diagram summarizing the systematic literature search is shown in Fig. 1.

**Table 1:** Characteristics of included studies

Author	Study year	Study type^a^	Study Arms	Samples		Age	Gender	Running time
				MLC	SILC		(F/M)	
Chekan	2013	nRCS	3	1940	527	18-80	1888/579	2009-2010
Dabbagh	2015	RCT	2	20	20	< 50	29/11	2013-2014
Hosogi	2011	nRCS	3	26	31	18-77	44/13	2009-2010
Hu	2013	RCT	2	30	30	-	-	2011-2011
Lee	2010	RCT	2	35	35	23-84	42/28	2008-2009
Saad	2013	RCT	3	35	35	-	52/18	2010-2011

^a^RCT, randomized controlled study; nRCSs, non-randomized comparative study

Three studies [21–23] used a three-arm design and compared MLC, SILC and LC. The other three studies [18–20] employed a two-arm design and made comparison between MLC and SILC.

### Risk of bias of included studies

Four RCTs [18–21] included were assessed: according to the Cochrane Collaboration’s tool, the generation of allocation sequence was adequate in 3 (75%) trials; allocation concealment was adequate in 3 (75%) trials; blinding of participants and personnel was adequate in 1 (25%) trial; blinding of outcome assessment was dequate in 3 (75%) trials; and the follow-up data were adequate in 4 trials (100%); Outcome data were adequate in 1 trial (25%). Then 1 (25%) trial was considered to be of low quality (high risk) and the other 3 (75%) were considered of high quality (low risk). (Table 2). According to the NOS, the quality assessment of other two nRCSs was also listed in Table 2, with their scores being 7 (high quality) and 5 (low quality), respectively.

**Table 2:** Methodological quality of included studies

Author	Year	Study type^a^	Random sequence generation	Allocation concealment (selection bias)	Blinding of participants and personnel (performance bias)	Blinding of outcome assessment (detection bias)	Incomplete outcome data (attrition bias)	Selective reporting (reporting bias)	NOS score	Quality of studies included^b^
Dabbagh	2015	RCT	Low risk	Low risk	High risk	Low risk	Low risk	High risk	-	High quality
Hu	2013	RCT	High risk	High risk	High risk	High risk	Low risk	High risk	-	Low quality
Lee	2010	RCT	Low risk	Low risk	High risk	Low risk	Low risk	High risk	-	High quality
Saad	2013	RCT	Low risk	Low risk	Low risk	Low risk	Low risk	Low risk	-	High quality
Chenkan	2013	nRCS	-	-	-	-	-	-	7	High quality
Hosogi	2011	nRCS	-	-	-	-	-	-	5	Low quality

^a^RCT, randomized controlled trial; nRCS, non-randomized comparative study

^b^According to the Cochrane Collaboration’s tool, the quality of RCT was considered high for more than 3 risk factors were low; according to the Newcastle-Ottawa Scale (NOS), the quality of nRCS was considered high for the score > = 6

### Operating time

**Fig. 2:**
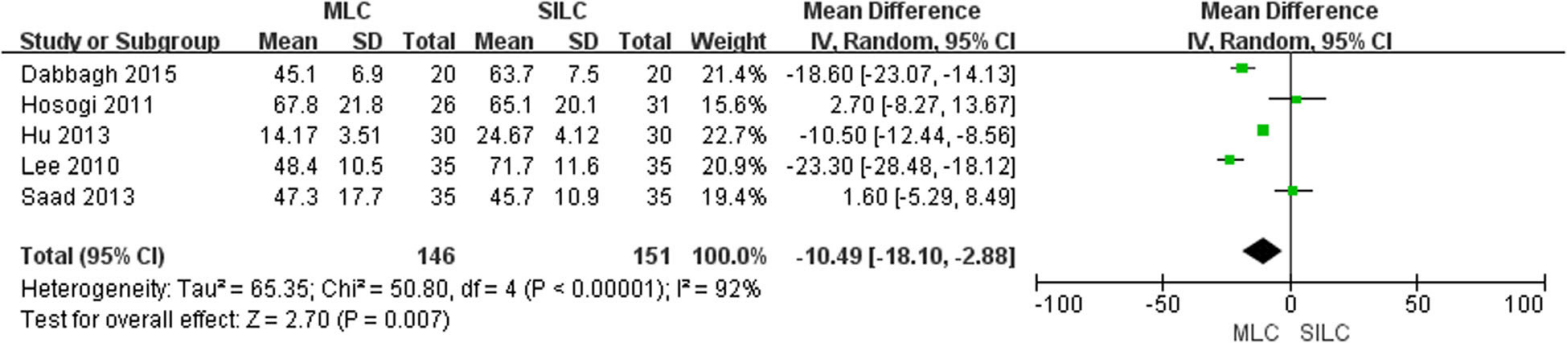
Forest plot on MLC vs. SILC in terms of operating time

Five included trials [18–21, 23] reported the operating time, and a significant difference in the random-effects model (MD -10.49; 95% CI -18.10, −2.88; *P* = 0.007) (Fig. 2). MLC required less operating time as compared with SILC. The funnel plot showed some biases that might result from exclusion or absence of some trials (left low part of the figure) (Additional file 2: Figure S1). Sensitivity analysis didn’t reveal any significant outlier (Additional file 2: Figure S2).

## Conversion

**Fig. 3:**
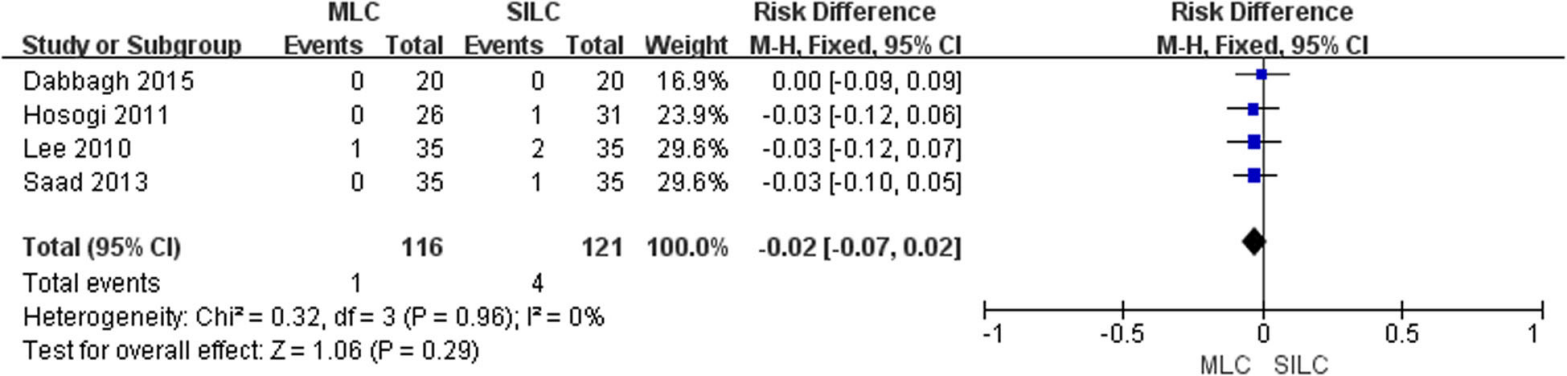
Forest plot on MLC vs SILC in terms of conversion

Four trials [18, 20, 21, 23] reported the conversion rate to LC. Although the conversion rate was lower in the MLC group, no statistical significance was found between the two procedures (RD -0.02; 95% CI -0.07, 0.02; *P* = 0.29) (Fig. 3). The causes for conversions mainly included poor visualization of the Calot’s triangle, densely fibrotic gallbladder and chronic cholecystitis. So, to a certain extent, technical innovation of SILC renders anatomical identification difficult. Funnel plot was symmetrical and there was no augmentation of bias (Additional file 2: Figure S3). Sensitivity analysis was not feasible because of zero conversion with both arms.

### Hospital stay

**Fig. 4:**
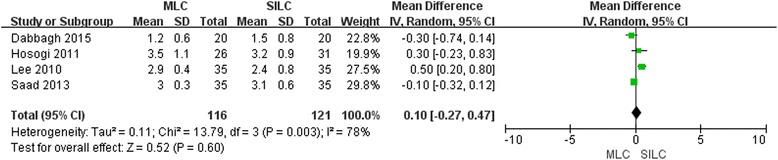
Forest plot on MLC vs SILC in terms of hospital stay

For trials [18, 20, 21, 23] reported the hospital stay and no significant difference (MD 0.10; 95% CI -0.27, 0.47; *P* = 0.60) was found between the two procedures and the heterogeneity was high (Fig. 4). The funnel plot was symmetrical and there was no augmentation of bias (Additional file 2: Figure S4). Sensitivity analysis didn’t reveal any significant outlier (Additional file 2: Figure S5).

### Complications

**Fig. 5:**
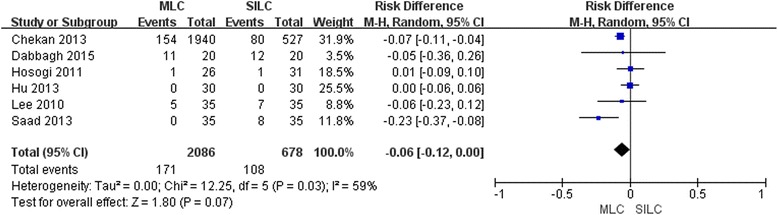
Forest plot on MLC vs SILC in terms of total complications

All 6 trials reported complications. No statistical significance (RD -0.06; 95% CI -0.12, 0.00; *P* = 0.07) (Fig. 5) was revealed in complications between the two procedures, although a lower incidence of total complications was found (8.2%, 171/2086) in MLC group than SILC group (15.9%, 108/678). The funnel plot exhibited some bias arising from the exclusion or absence of some trials (right low part) (Additional file 2: Figure S6). Sensitivity analysis could not be performed because of zero complications with both arms. All complications are listed in Table 3. The most commonly reported complications were nausea and vomiting, with a morbidity of 4.5% in MLC and 8% in SILC, respectively.

**Table 3:** Total complications and proportions

Complications	MLC	SILC
bile duct obstrction	2 (0.1%)	2 (0.3%)
cerebrovascular accidents	1 (0.0%)	0 (0.0%)
acute myocardial infarction	2 (0.1%)	0 (0.0%)
transient ischemic attack	2 (0.1%)	0 (0.0%)
other embolisms	3 (0.1%)	0 (0.0%)
pulmonary embolism	3 (0.1%)	1 (0.1%)
digestive system complications	15 (0.7%)	8 (1.2%)
gastroparesis paralytic ileus	7 (0.3%)	4 (0.6%)
nausea and vomiting	93 (4.5%)	54 (8.0%)
operative complications	30 (1.4%)	19 (2.8%)
peritonitis	3 (0.1%)	1 (0.1%)
bile spillage	9 (0.4%)	9 (1.3%)
urinary retention	1 (0.0%)	1 (0.1%)
peforation of diaphragm	0 (0.0%)	1 (0.1%)
wound infection	0 (0.0%)	6 (0.9%)
incisional hernia	0 (0.0%)	1 (0.1%)
choledocholithiasis	0 (0.0%)	1 (0.1%)
Total	171 (8.2%)	108 (15.9%)

### Pain relief

**Fig. 6:**
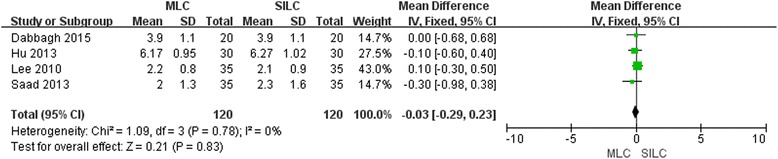
Forest plot on MLC vs SILC considering pain relief

Five studies [18–21, 23] reported pain relief, subjectively rated by the patients on a visual analogue scale of 0-10 points (from no pain to worst one). One study [23] reported the pain score by means of a diagram without presenting specific data. Then we compared the pain scores of the other 4 studies and found no significant difference (MD -0.03; 95% CI -0.29, 0.23; *P* = 0.83) in pain relief between the two procedures (Fig. 6). The funnel plot was symmetrical and there was no augmentation of bias (Additional file 2: Figure S7). Sensitivity analysis didn’t reveal any significant outlier (Additional file 2: Figure S8.).

### Cosmetic score and wound length

**Fig. 7:**
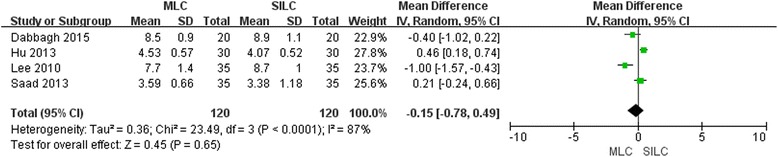
Forest plot on MLC vs SILC considering cosmetic score

**Fig. 8:**
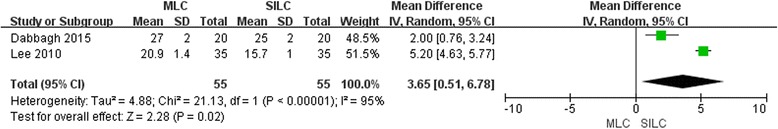
Forest plot on MLC vs SILC considering wound length

Five studies [18–21, 23] reported cosmetic results. One of the studies [23] reported cosmetic results in terms of the number of the patients who were satisfied with their wounds and the other four studies evaluated with a visual analogue scale. Then the remaining four studies were pooled together for evaluation of cosmetic results and no significant difference (MD -0.15; 95% CI -0.78, 0.49; *P* = 0.65) was found (Fig. 7). Two studies [18, 20] also reported the wound length after the operation and a longer (MD 3.65; 95% CI 0.51, 6.78; *P* = 0.02) wound length was found in MLC (Fig. 8). So the wound length was not the only consideration in cosmetic evaluation. The funnel plot and sensitivity analysis were not performed due to too small a sample size.

## Discussion

MLC and SILC are two major technical innovations since introduction of LC, and are designed to reduce the invasiveness. Nonetheless, studies that directly compared the two procedures were scanty. This meta-analysis, for the first time, pooled the studies comparing MLC and SILC and examined their advantages and drawbacks. Our study showed that that: (1) MLC was associated with a shorter operating time but a longer wound length as compared with SILC; (2) there existed no significant differences in hospital stay, pain relief, cosmetic result and complications between the two procedures; (3) MLC had lower conversion and complication rates than SILC but the differences were not statistically significant.

This meta-analysis revealed that MLC had a significantly shorter operating time when compared to SILC. MLC uses of smaller diameter instruments, compared with LC which employs 5-mm instruments and it keeps original triangulation and can achieve satisfactory retraction. But SILC is an unnaturally ergonomic technique for both the operative instruments and camera are placed together virtually into the same incision and on the same axis. As a result, the traditional triangulation is lost, which may contribute to longer operating time with SILC. In fact, operating time was reportedly longer with SILC than with LC due to the loss of traditional triangulation [24]. In addition, it was reported that the the surgeon’s experience [25] could be responsible for the longer operating time in SILC. But we could not evaluate its influence on the operating time since very few studies covered the surgeon’s experience.

The loss of triangulation in SILC also led to a high conversion rate, especially in complicated scenarios, such as, chronic cholecystitis with densely fibrotic gallbladder. But in this study, no statistical significance in conversion rate was found between MLC and SILC, although a relatively lower conversion rate was found in MLC (0.9%) compared to SILC (3.3%). It was reported the addition of at least one port was necessary with SILC in about 5% to 8.4% of cases [7, 26].

No significant difference was found in hospital stay between MLC and SILC. Hospital stay was always a key measure reflecting patients’ convalescence. A shorter hospital stay could be the result of fewer complications and a faster recovery. Recently, due to the implementation of fast-tract pathways for LC [27], day-case procedures have been increasingly accepted for its cost-effectiveness. Then patients are discharged practically within 24 h or even the day of surgery, which makes the hospital stay a meaningless indicator of patients’ convalescence. In addition, return to work or activity was also taken as a measure of patients’ convalescence. However, in this study we could not perform the analysis for only one study [20] assessed the outcome and no difference was found in time taken to return to work between MLC and SILC.

Any innovation of the surgical technique is aimed at minimizing post-operative trauma and the pain. It was reported that better pain relief was achieved with SILC than with LC [28, 29] and pain degree was comparable with both MLC and LC [29]. Nevertheless, this study failed to reveal any significant difference in pain relief between MLC and SILC, which might be ascribed to the absence of unified criteria for pain evaluation and to differences in clinical practice. Since most studies included in this meta-analysis only covered certain aspects, selection and reporting biases were very likely.

As for cosmetic results, our analysis demonstrated SILC yielded a better cosmetic outcome as compared to LC [30], and this difference was reportedly explained [20] by the shorter wound length associated with SILC. However, our study showed that, although SILC had a significantly shorter wound length, but no significant difference was revealed in cosmetic outcome between the two procedures. We are led to believe that the wound length might not be the sole consideration of patients that determined their satisfaction with incision.

There was no statistically significant difference in complications rate between the two procedures, although a low incidence of total complications was found with MLC (8.2%) than with SILC (15.9%). This outcome was inconsistent with a previous finding that [21] complication rate was higher in SILC than in MLC and LC. Moreover, another study, involving 1000 cases of cholecystectomy in 427 settings [22], reported a significantly higher incidence of complications with SILC than with MLC and LC, especially in terms of postoperative nausea and vomiting, digestive system complications, and operative complications. One possible explanation for the discrepancy might be the small sample size of RCTs, which might increase the reporting and selecting biases [31]. Therefore, further meta-analyses involving more RCTs are warranted to confirm our results.

This study had some limitations. First, only a small number of studies were included and we were unable to make comparison between RCTs and nRCSs, high risk trials and low risk ones, which might cause selecting bias. Second, 83.3% (5/6) of the studies involved no more than 35 cases for each arm, which might bring about reporting bias.

## Conclusion

MLC has an advantage over SILC in terms of operating time rather than hospital stay, pain relief, cosmetic results. Conversion and complication rates, though higher in SILC, were not statistically different between the two procedures. Whether MLC confers any benefits in conversion or complication rates still needs further research.

## Additional files

Additional file 1: NEWCASTLE - OTTAWA QUALITY ASSESSMENT SCALE (DOCX 14 kb)

Additional file 2: Figure S1. Funel plot on MLC vs SILC considering operating time. **Figure S2.** Sensitivity analysis on MLC vs SILC considering operating time. **Figure S3.** Funel plot on MLC vs SILC considering conversion. **Figure S4.** Funel plot on MLC vs SILC considering hospital stay. **Figure S5.** Sensitivity analysis on MLC vs SILC considering hospital stay. **Figure S6.** Funel plot on MLC vs SILC considering total complications. **Figure S7.** Funel plot on MLC vs SILC considering pain. **Figure S8.** Sensitivity analysis on MLC vs SILC considering pain. (PDF 949 kb)

## Abbreviations

CI: Confidence interval
LC: Laparoscopic cholecystectomy
MD: Mean difference
MLC: Mini-laparoscopic cholecystectomy
nRCS: Non-randomized comparative study
RCT: Randomized controlled trial
RD: Risk difference
SILC: Single-port laparoscopic cholecystectomy
