# Supplementary material for: Minilaparoscopic versus single incision cholecystectomy for the treatment of cholecystolithiasis: a meta-analysis and systematic review
Source: BMC Surg. 2017 Aug 22;17:91. doi: 10.1186/s12893-017-0287-x (PMC5568361; doi:10.1186/s12893-017-0287-x)

Figure S1. Funel plot on MLC vs SILC considering operating time.

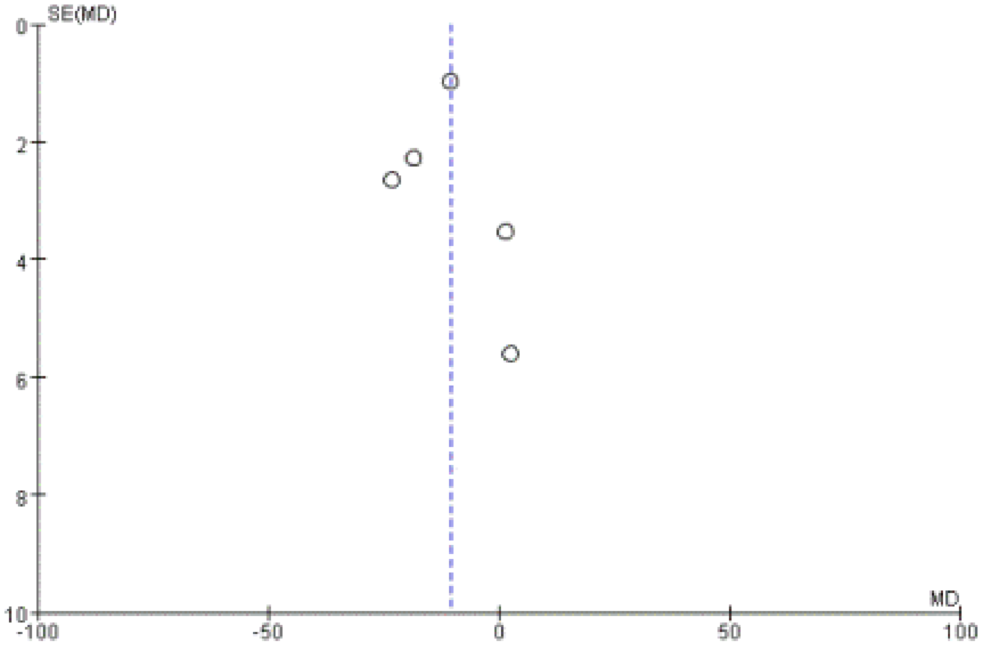

Figure S2. Sensitivity analysis on MLC vs SILC considering operating time.

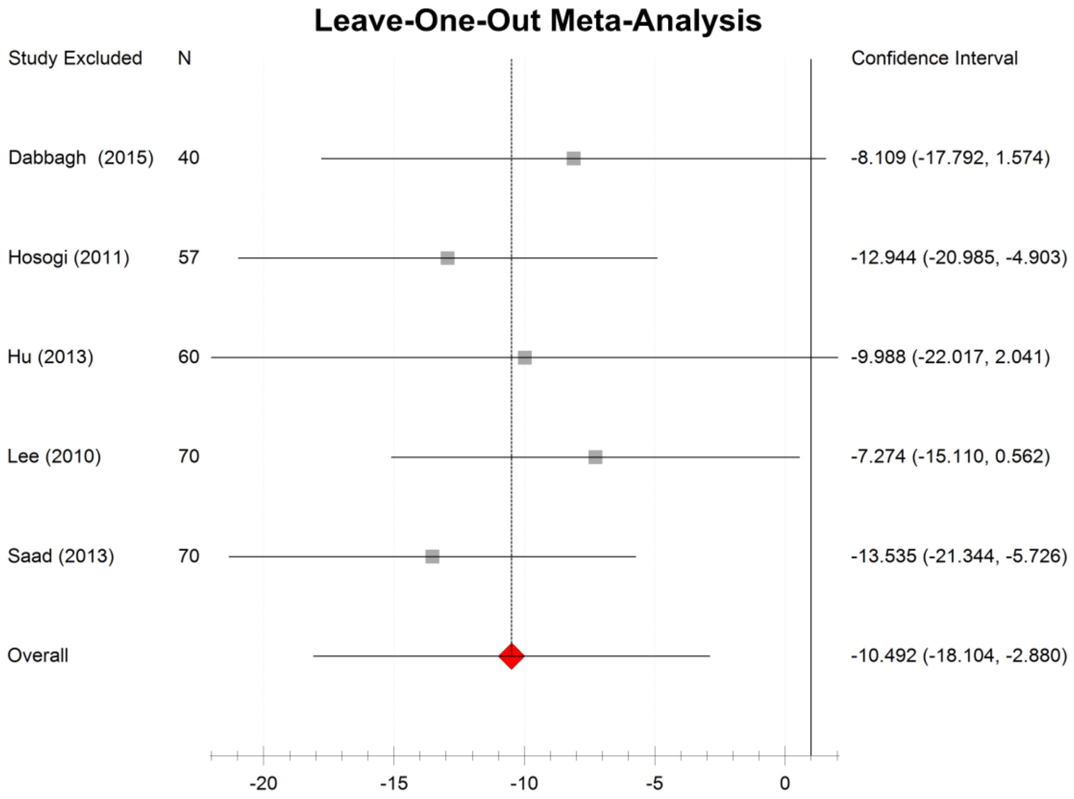

Figure S3. Funel plot on MLC vs SILC considering conversion.

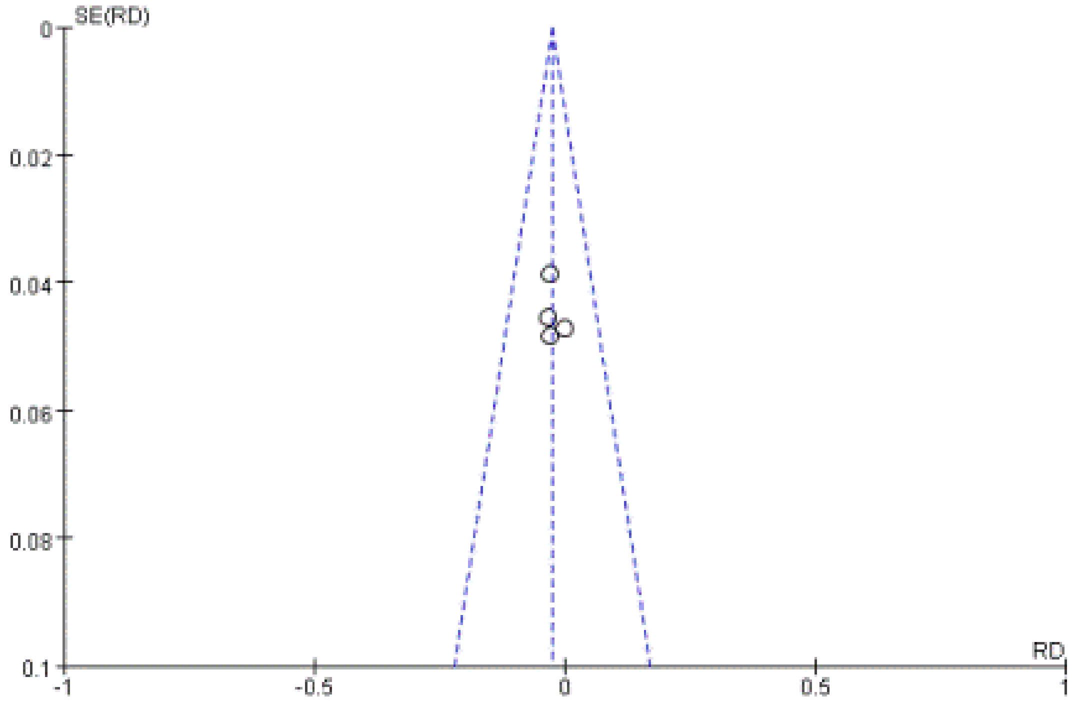

Figure S4. Funel plot on MLC vs SILC considering hospital stay.

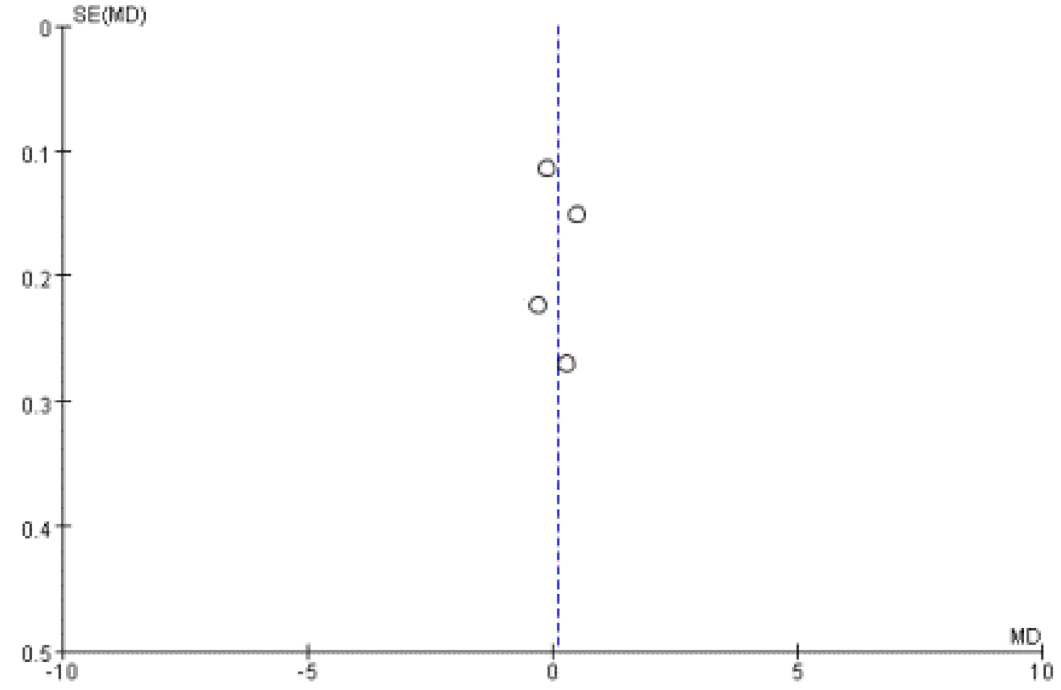

Figure S5. Sensitivity analysis on MLC vs SILC considering hospital stay.

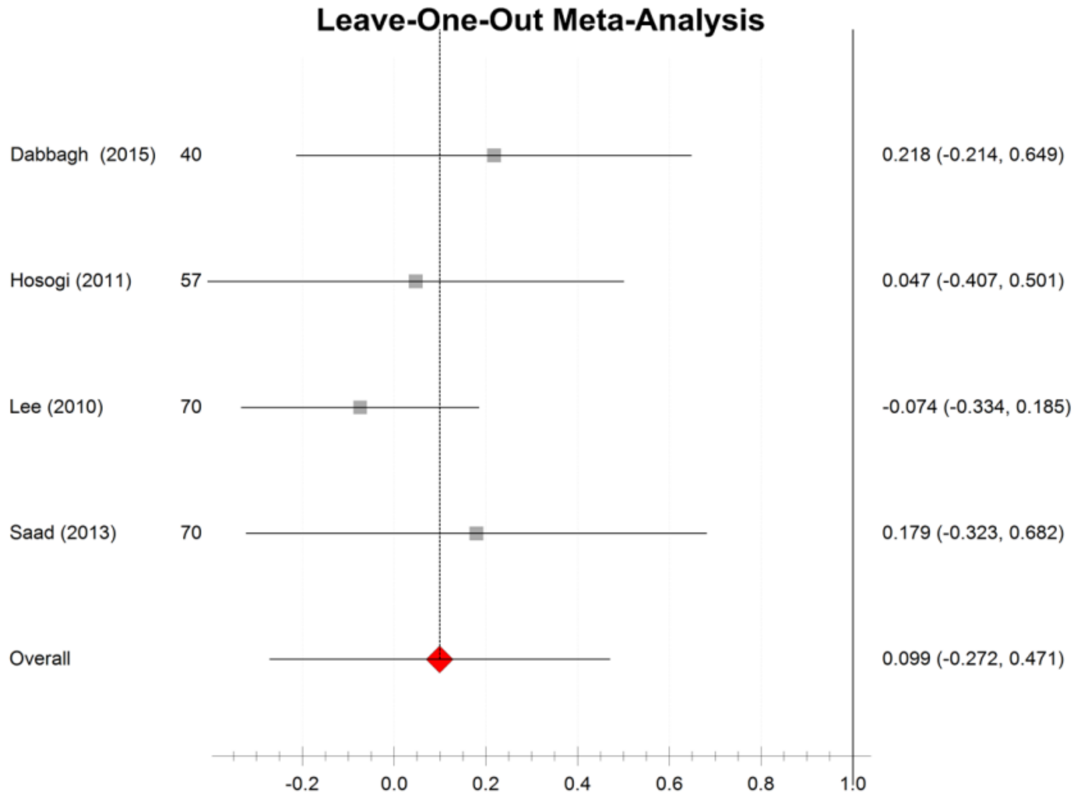

Figure S6. Funel plot on MLC vs SILC considering total complications.

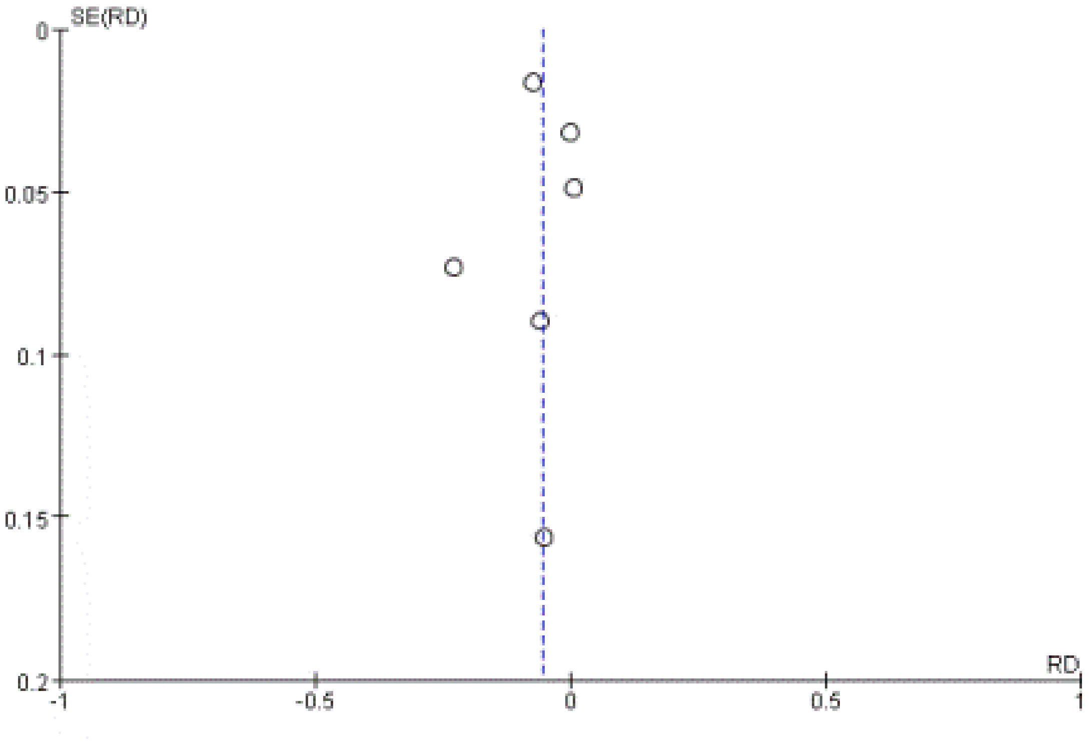

Figure S7. Funel plot on MLC vs SILC considering pain.

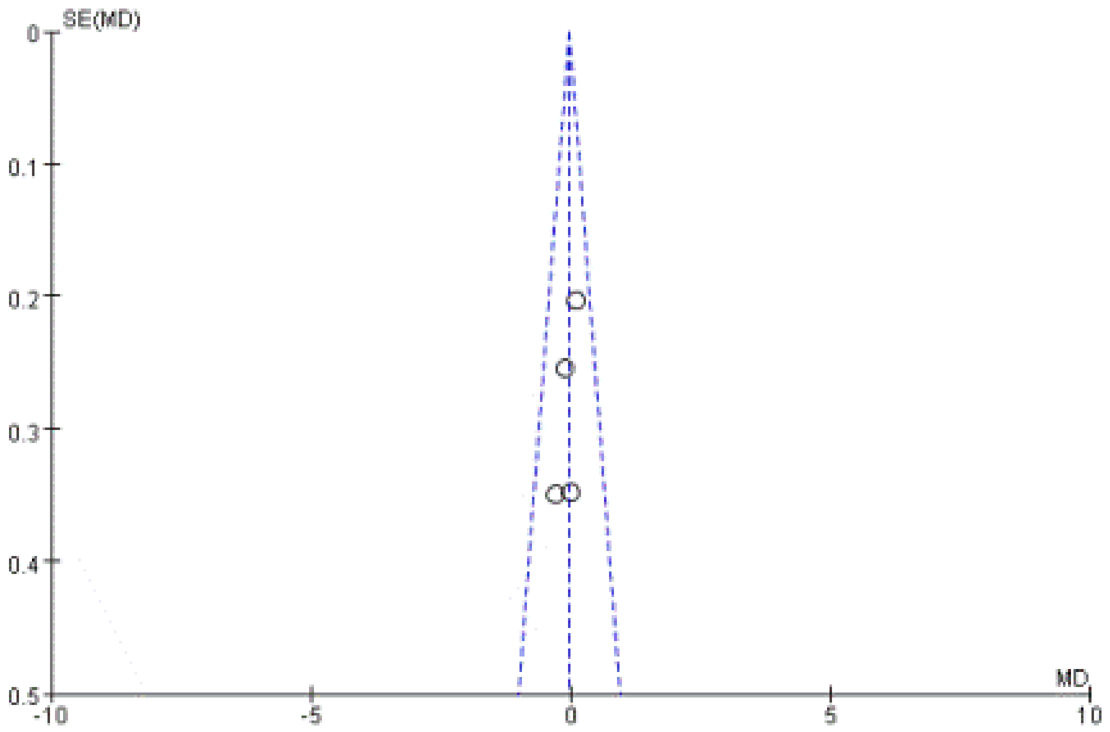

Figure S8. Sensitivity analysis on MLC vs SILC considering pain.

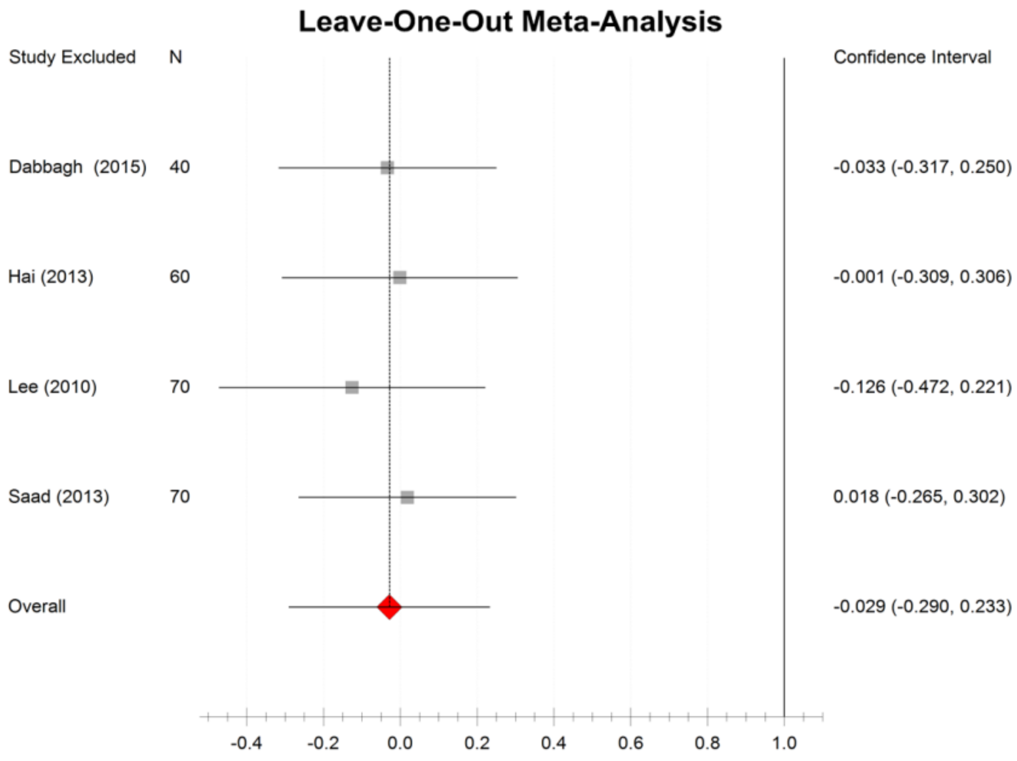

Supplement: Supplementary file 2 — Funel plot on MLC vs SILC considering operating time. Figure S2. Sensitivity analysis on MLC vs SILC considering operating time. Figure S3. Funel plot on MLC vs SILC considering conversion. Figure S4. Funel plot on MLC vs SILC considering hospital stay. Figure S5. Sensitivity analysis on MLC vs SILC considering hospital stay. Figure S6. Funel plot on MLC vs SILC considering total complications. Figure S7. Funel plot on MLC vs SILC considering pain. Figure S8. Sensitivity analysis on MLC vs SILC considering pain. (PDF 949 kb) [file 12893_2017_287_MOESM2_ESM.pdf]
